# Wind-assisted high-altitude dispersal of mosquitoes and other insects in East Africa

**DOI:** 10.1093/jme/tjad033

**Published:** 2023-04-24

**Authors:** Harrysone E Atieli, Guofa Zhou, Daibin Zhong, Xiaoming Wang, Ming-chieh Lee, Alpha S Yaro, Moussa Diallo, John Githure, James Kazura, Tovi Lehmann, Guiyun Yan

**Affiliations:** Sub-Saharan International Center of Excellence for Malaria Research, Tom Mboya University, Homa Bay, Kenya; Program in Public Health, University of California, Irvine, CA, USA; Program in Public Health, University of California, Irvine, CA, USA; Program in Public Health, University of California, Irvine, CA, USA; Program in Public Health, University of California, Irvine, CA, USA; Malaria Research and Training Center (MRTC)/Faculty of Medicine, Pharmacy and Odonto-Stomatology, Bamako, Mali; Malaria Research and Training Center (MRTC)/Faculty of Medicine, Pharmacy and Odonto-Stomatology, Bamako, Mali; Sub-Saharan International Center of Excellence for Malaria Research, Tom Mboya University, Homa Bay, Kenya; Center for Global Health and Diseases, School of Medicine, Case Western Reserve University, Cleveland, OH, USA; Laboratory of Malaria and Vector Research, NIAID, NIH, Rockville, MD, USA; Program in Public Health, University of California, Irvine, CA, USA

**Keywords:** altitude, migration, mosquito, insect, Africa

## Abstract

Knowledge of insect dispersal is relevant to the control of agricultural pests, vector-borne transmission of human and veterinary pathogens, and insect biodiversity. Previous studies in a malaria endemic area of the Sahel region in West Africa revealed high-altitude, long-distance migration of insects and various mosquito species. The objective of the current study was to assess whether similar behavior is exhibited by mosquitoes and other insects around the Lake Victoria basin region of Kenya in East Africa. Insects were sampled monthly from dusk to dawn over 1 year using sticky nets suspended on a tethered helium-filled balloon. A total of 17,883 insects were caught on nets tethered at 90, 120, and 160 m above ground level; 818 insects were caught in control nets. Small insects (<0.5 cm, *n* = 15,250) were predominant regardless of height compared with large insects (>0.5 cm, *n* = 2,334) and mosquitoes (*n* = 299). Seven orders were identified; dipteran was the most common. Barcoding molecular assays of 184 mosquitoes identified 7 genera, with *Culex* being the most common (65.8%) and *Anopheles* being the least common (5.4%). The survival rate of mosquitoes, experimentally exposed to high-altitude overnight, was significantly lower than controls maintained in the laboratory (19% vs. 85%). There were no significant differences in mosquito survival and oviposition rate according to capture height. These data suggest that windborne dispersal activity of mosquito vectors of malaria and other diseases occurs on a broad scale in sub-Saharan Africa.

## Background

A universally accepted definition of animal migration that cuts across all animal species have proved to be a challenge to ecologists (Dingle 2014). For decades, insects’ movement has elicited varying opinions among entomologists of the precise definitions of “migration” and “dispersal” (Johnson 1960, Baker et al. 1980, Service 1993, Lincoln 1998). Indeed, some pundits suggest that insect migration can be broadly classified into 3 types: (i) migration on one-way journeys for the purpose of breeding; (ii) migration from a breeding area to a feeding area; and (iii) migration from a breeding area to hibernation or estivation sites (Gao et al. 2020). Integrating insects’ migration into spatial population dynamics has opened the field of dispersal ecology (Clobert et al. 2009, Stevens et al. 2012, 2014). A section of dispersal ecologists describes migration briefly as round-trip animal movement between their regular breeding and nonbreeding habitats (Clobert et al. 2009).

Given the diversity and complexity in purpose and process of insect movement, we adopted a broader definition based on the principle that migration is a persistent straightened-out movement that is based on behavior that is undistracted by cues, which would otherwise arrest other types of movement (e.g., attraction to host, mate, or oviposition site) and which is expected to land the individual in a distant environment with distinct resources (Dingle and Drake 2007, Dingle 2014). This approach has a strength of focusing on both the behavioral and ecological mechanisms underlying the movement pathways and outcomes, respectively (Nathan et al. 2008).

Of public health importance, mosquito dispersal strongly affects vector population dynamics and malaria transmission risk and the effectiveness of malaria vector control interventions. Conventional studies on the dispersal of mosquito vectors used the mark–release–recapture technique. This technique is suitable for studying short-distance dispersal required by mosquitoes to find mates, sugar-feeding sources, suitable resting sites, breeding habitats, and blood meal sources. Wind-assisted long-distance dispersal of mosquitoes has long been suspected, but empirical evidence for this mode of movement is lacking due to the absence of suitable research tools. Earlier studies by Service (1997) preferred describing all flights by mosquitoes as wind assisted, or otherwise as dispersal, because there was no convincing evidence that mosquitoes purposely fly high to be swept away to colonize new territories or, that they or their progeny, return to their original areas. However, a few mosquito species such as *Aedes taeniorhynchus* and *Anopheles pharoensis* are sometimes described as migratory (Provost 1952). The majority of insect migration has been documented in the subtropics (Chapman et al. 2015, Florio et al. 2020). Surprisingly, a regular, large-scale windborne mosquito migration at high altitude was reported recently above the Sahel of West Africa (Huestis et al. 2019, Florio et al. 2020). As an extremely seasonal environment with abundant resources in the wet season, the Sahel is expected to be a hotbed for migrants, yet it remains unclear if mosquitoes residing at perennial environments, hundreds of kilometers away from seasonal habitats, also engage in windborne migration. To determine whether mosquitoes and other insects engage in high-altitude dispersal activities in equatorial regions of East Africa, the current study therefore deployed an aerial sampling technique like the one used by Huestis et al. (2019).

Using helium-inflated balloons and sticky insect traps, this study demonstrated high-altitude, long-distance dispersal of various mosquito species, including *Anopheles*, *Aedes*, and *Culex* mosquitoes species. Similar to observations made in the Sahel, mosquitoes collected at 90–160 m above ground level (agl) in Equatorial East Africa were dominated by gravid females, indicating that they have been exposed to at least one blood meal and may carry pathogens. This new aerial sampling technique provides fresh insights into mosquito behavior and may change the prevailing view on short-distance dispersal by disease vectors.

## Materials and Methods

### Study Location

**Fig. 1.**
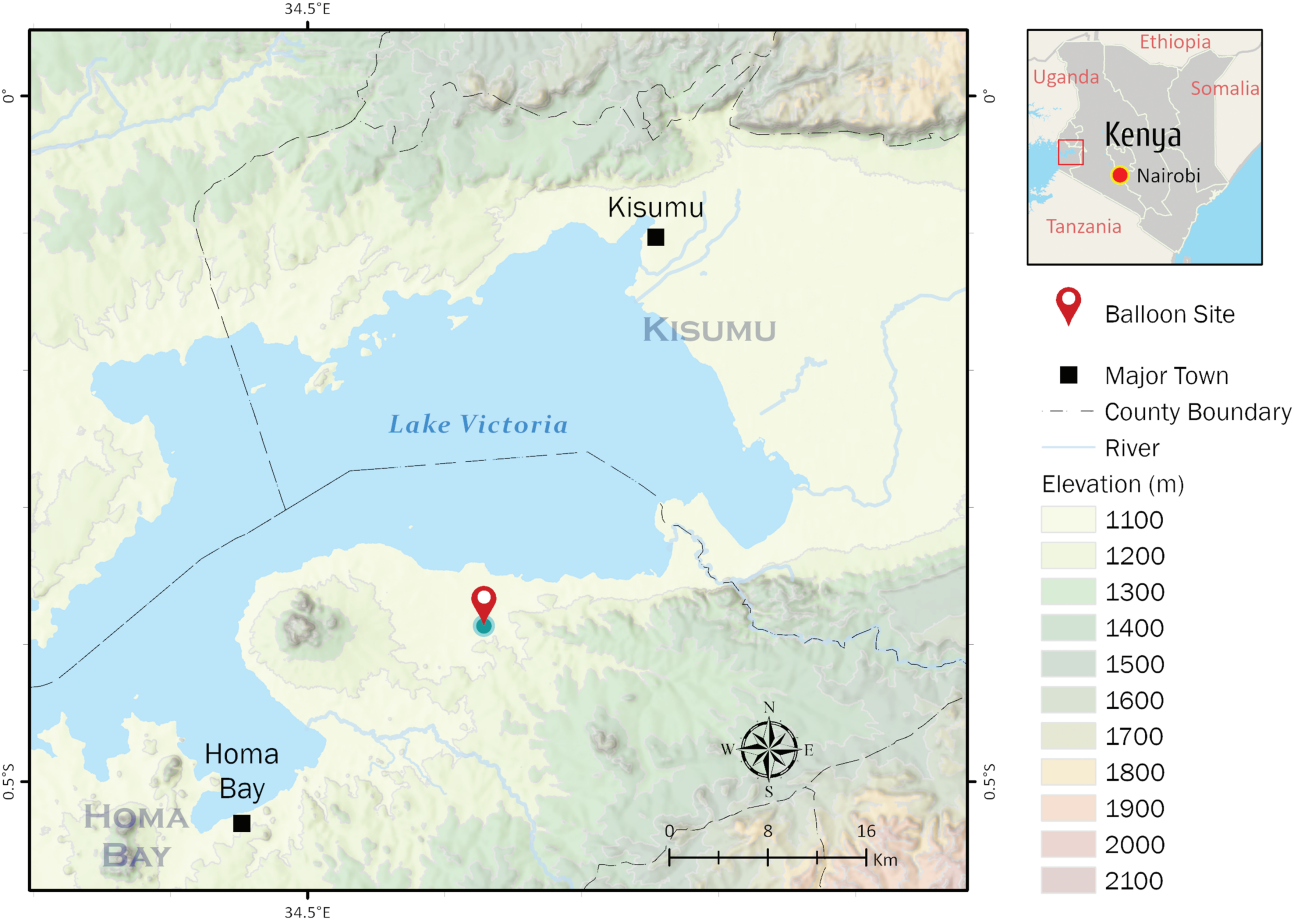
Study site.

The study was conducted in Homa Bay County, western Kenya, between December 2018 and December 2019. Homa Bay County experiences tropical climatic condition. It is classified as a lowland with low topographic landscape. It is situated along the southern shores of Winam Gulf, northeastern corner of Lake Victoria (34.6°E and 0.5°S; 1,330 m asl) (Fig. 1). The average annual temperature in this region is 22.5 °C (72.6 °F) with an annual rainfall of 1,646 mm (64.8 inch). There are 2 rain seasons per year—from March to May and from September to November. Hourly average wind speed varies within the year. The most windy part of the year lasts for 3–4 months between December and March with an average wind speed of >10.5 km/h. The wind direction is most often from the east (easterly wind) for about 11 months from April to March with a peak percentage of 38%, except for 1 month (March–April), when easterly wind prevails with a peak percentage of 35%. Agricultural activities in Homa Bay County rely mainly on rain-fed small-scale farming and an irrigation project known as the Kimira and Oluch Irrigation Scheme. The dominant farming crops are rice, maize, and vegetables. Given that this region is classified as semiarid, the irrigation scheme was established following frequent crop failures resulting in food insecurity. The establishment of this irrigation infrastructure has boosted land productivity through the production of both horticulture and staple foods and substantially increased incomes for about 3,000 households within the irrigation scheme. Conversely, the development project has its associated shortcomings, such as increased aquatic habitats for malaria vector breeding and introduction of agricultural pests. Malaria transmission is endemic in this region with noticeable transmission peaks during the rainy seasons. *Plasmodium falciparum* accounts for the majority of malaria cases in this region (Bousema and Baidjoe 2013, Bousema et al. 2013).

### Field Balloon Launch and Aerial Insects Sampling

**Fig. 2.**
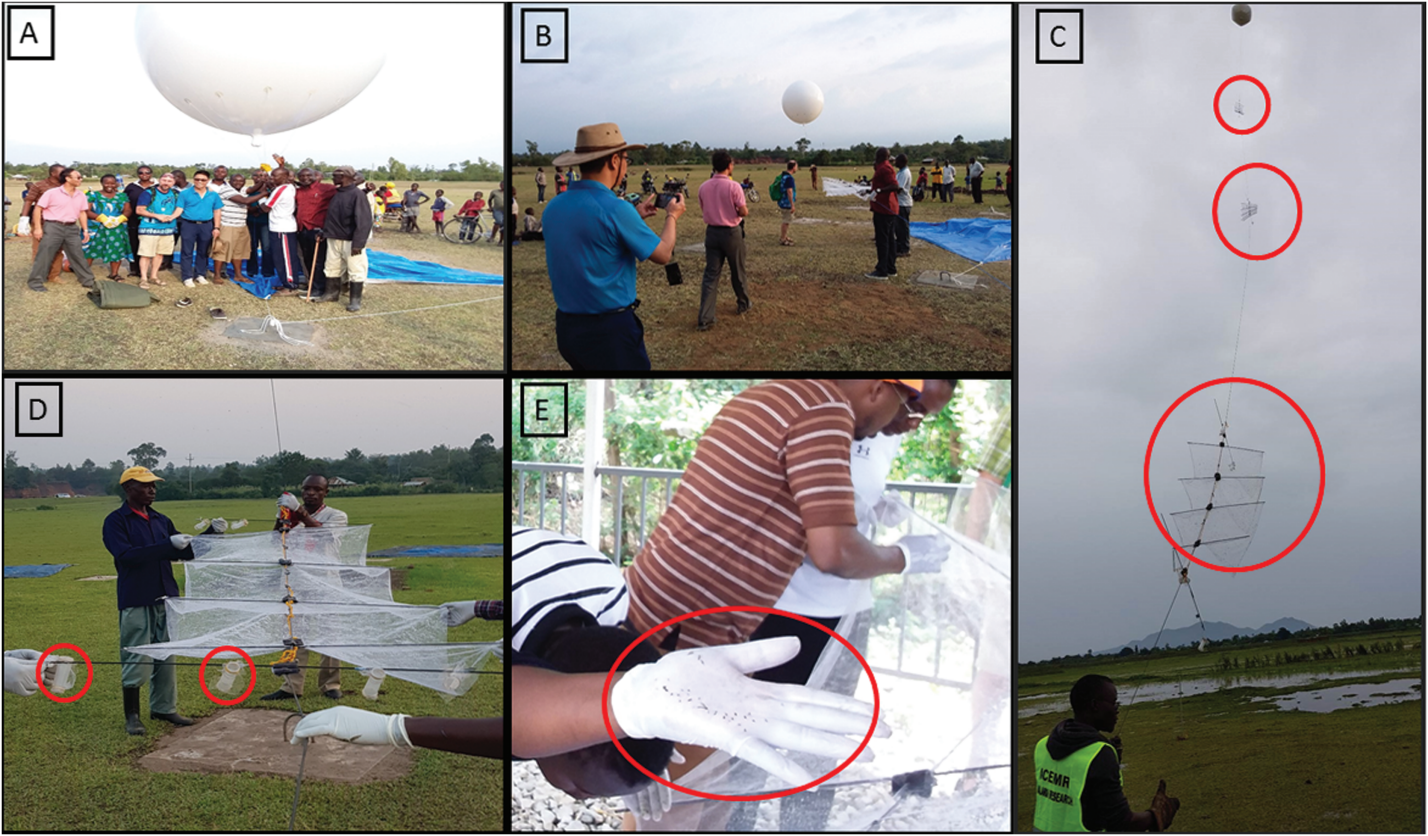
A) Launching tethered balloon. B) Fixing the sticky panels on the tethered string. C) Tethered balloon with 3 sticky panels at heights 9, 120, and 160 m agl. D) Sticky panel and open ended suspended falcon tubes containing mosquitoes exposed to high-altitude condition. E) Collecting and sorting captured insects.

Aerial sticky net sampling was carried out at ~160 m agl at an open field site near the shore of Lake Victoria (34.37°E and 0.23°S; 1,120 m above sea level) following permission and authorization from the Kenya Civil Aviation Authority. As described in detail in previous studies that focused on *Anopheles* mosquitoes in the Sahel (Huestis et al. 2019), briefly, rectangular 3 × 1 m^2^ nets were stitched to form sleeves of 1 m apart along the net with hollow light-weight carbon rods inserted and attached to the net using Duco cement glue. Nets were coated with a thin film of insect glue to form a sticky net referred to as a panel. Prior to launch, 3-m-diameter polyurethane balloons were inflated to capacity with balloon-grade helium (Fig. 2). This was topped off in subsequent days to ensure full capacity. With an exception of first month 8-day collection, at each sampling point, 10 consecutive day aerial insect sampling collections were performed each month for 12 months using sticky net panel traps attached to the tethering line of the balloon, with each balloon typically suspending 3 panel traps. Panel traps were suspended at 90, 120, and 160 m agl as standardized by previous studies in Mali (Huestis et al. 2019, Florio et al. 2020). The balloon was kept stationary at 200 m agl by a cord secured to a cement block buried in the ground. Between launches, the balloon was secured over a “landing patch” padded by tires covered by a tarp. A 5-person team provided 24-h maintenance and site coverage for the duration of the sampling. Another 2-person team provided daily support to collect, label, and preserve trapped specimens.

The sticky panel traps suspended on the balloon were launched in the evening 1 h before sunset (~17:00 h) and retrieved 1 h after sunrise (~07:30 h) the following morning. The choice of night sampling was based on previous literature especially based on radar showing that small insects use the lower jet stream that flows over the temperature inversion layer, which develops at night (Drake and Reynolds 2012). For quality control and to minimize bias from insects trapped near the ground, comparable panels were raised up to 100 and 120 m agl and immediately retrieved during each trapping launch and retrieval operation. Retrieved panels were inspected for insects in a dedicated clean wind-free area to minimize contamination. Individual insects were collected carefully from the sticky nets using forceps, counted, and preserved in labeled vials containing 80% ethanol. All trapped mosquitos and other insects were identified to order, family, or genus level by morphology. All mosquitoes were preserved in individual Eppendorf tubes with RNAlater and processed for molecular identification and pathogen detection, whereas all other insects were preserved in ethanol.

### Mosquito Molecular Analysis

Sampled insects stained with glue were cleaned carefully using 100% chloroform. Individual cleaned mosquitoes were preserved in 1.5-ml Eppendorf tube filled with RNAlater. A dissecting microscope was used for morphological identification of individual mosquitoes using the Gillies and Coetzee taxonomic keys (Gillies and Coetzee 1987). Genomic DNA and total RNA of individual specimens were isolated and purified using the Quick-DNA/RNA Microprep Plus Kit (Zymo Research, Irvine, CA) according to the manufacturer’s instructions and maintained at −80 °C until further use. PCR amplification of the target DNA barcode region (658 bp) was performed using forward primer LCO1490 (5ʹ-GGT CAA CAA ATC ATA AAG ATA TTG G-3ʹ) and reverse primer HCO2198 (5ʹTAA ACT TCA GGG TGA CCA AAA AAT CA-3ʹ) (Folmer et al. 1994). PCR was carried out in total reaction volume of 17 μl that included 1 μl of DNA template (~5 ng), 5 pmol of each primer, and 8.5 μl of DreamTaq Green PCR Master Mix (2×) (Thermo Fisher Scientific, Waltham, MA). The PCR cycling conditions were as follows: an initial denaturation step at 94 °C for 3 min followed by 35 cycles of 94 °C for 30 s, 55 °C for 30 s, 72 °C for 1 min, and a final extension step at 72 °C for 6 min. Positive and negative controls for the amplification reactions were carried out at every PCR round for quality control. The PCR products were confirmed by gel electrophoresis on a 2% agarose gels and purified using an enzymatic PCR clean-up technique. Sanger sequencing was performed bidirectionally using the same primers as used in PCR amplifications by GENEWIZ, Inc. (South Plainfield, NJ). Multiplex PCR was conducted to distinguish between *An. gambiae* s.l. sibling of *An. gambiae* s.s. and *An. arabiensis* according to the method described by Scott et al. (1993). Detection of flaviviruses and alphaviruses was performed using reverse transcription (RT-PCR) with the universal primer pairs for flaviviruses (Scaramozzino et al. 2001) and alphaviruses (Sanchez-Seco et al. 2001).

### Molecular Identification and Phylogenetic Analysis of Specimens

In this study, a molecular identification method, known as Codon Code Aligner 9.0.1 (Codon Code Corporation, Centerville, MA), was used to check the sequence quality and trim low-quality bases, while BioEdit software was used to align the sequences and to calculate pairwise sequence identity and similarity from multiple sequence alignments. A 98% threshold limit of the sequence similarity was used to classify sequences into species groups, whereas the consensus sequences within group were compared with the BOLD database (https://www.barcodinglife.org) (Ratnasingham and Hebert 2007). To confirm species and examine their evolutionary relationship, phylogenetic analyses were performed using UPGMA with the Kimura 2-parameter model for COX1 haplotypes in the MEGA version 7.05 (Kumar et al. 2016). The phylogenetic tree nodes were evaluated by bootstrap analysis for 1,000 replicates using reference sequences from GenBank (KU380473, KU380462, KU380457, KU380471, KU187054, KU187087, KU380423, KU380445, LC473638, KU380397, GQ165804, MK533634, KJ940558, KJ940551, KJ940657, KU380404, MK533643, KU380466, KT382816, GQ165800, KU187159, MT598105, LC473710, GQ165789, LC473708, JN298324, JN291712, KU186990, KU187113, KU380406, KY670610).

### High-Altitude Mosquito Survival Assays

Laboratory-reared fully gravid female *An. gambiae* s.s. of age 5 days were randomly assigned to altitude exposure treatments at 90, 120, and 160 m agl and allowed to stay overnight, that is, for 14.5 h. Each female mosquito was placed in a 50-ml Falcon tube measuring 5 cm long and 3 cm diameter as described in previous studies optimized for a similar experiment in Mali (Sanogo et al. 2021). Both sides of the tube were cut open and covered with a netting material with holes measuring 1.5 mm to allow ventilation and natural airflow. Ten tubes each containing one mosquito were suspended on each panel using a string at every height and left to stay overnight (10 mosquitoes/panel/height/night). The balloon was launched in the evening approximately 1 h before sunset (~17:00 h) and retrieved the following morning 1 h after sunrise (~07:30 h). This exposure was replicated 11 consecutive nights with varied climatic conditions in the month of November–December. Insectary-reared mosquitoes of the same age and from the same colony were used as controls. One hour upon retrieval in the morning, mosquitoes were examined for mobility and recorded as live (mobile) or dead (immobile). Live retrieved mosquitoes were delicately transported back to the insectary situated 10 km from the sampling site and further subjected to oviposition assay.

### Mosquito Oviposition Assay

Live mosquitoes that completed survival assays were transferred to the insectary and placed in a miniature cage. An individual laying pad made of wet cotton wool spread in a petri dish and covered with a filter paper on top was placed in each cage to quantify oviposition. A 10% sugar solution cotton ball was provided for feeding. Each cage and laying pad was monitored every morning for eggs until the mosquito died, and the number of eggs laid per day per mosquito was recorded.

### Statistical Analysis

Differences in the number of mosquitoes, number of large and small insects, and total number of insects collected at different heights were compared using Kruskal–Wallis rank tests, analysis of variance (ANOVA), and post hoc Tukey Honest Significant Difference (HSD) test were used for pairwise comparisons at a significant level of 0.05. One-way ANOVA was used to compare the differences in number of different types of insects (mosquitoes, small and large insects) at the same collection height using log(*x*+1) transformed counts, and paired *t*-test was used for the pairwise comparisons. The χ^2^-test was used to examine differences in insect species composition and differences in female mosquito oviposition rate between different collection heights. One-way ANOVA was also used to examine the differences in the number of eggs produced per female between experiments, and Tukey HSD test was used to compare pairwise differences between any 2 experiments with a significance level of 0.05.

## Results

### The Effect of Height on Insect Collection

A total of 384 experimental net panels were surveyed in 128 nights of aerial sampling from December 2018 to December 2019. A total of 17,883 insects were collected at 90, 120, and 160 m agl (Table 1). The average number of insects collected per netting panel was 46.6 insects at the 3 different heights. Eight hundred and eighteen insects were collected in a total of 256 control panels during the same sampling period, with an average of 3.19 insects per panel. Table 1 shows the total catch numbers categorized as mosquitoes and other insects ranked according to their size.

**Table 1.** Summary of aerial insects sampled at different heights (*N* = 128 panels at each height)

Height (m)	90	120	160	Total (%)	Control (*N* = 256)
Mosquitoes (%)	141 (2.2)	100 (1.7)	58 (1.1)	299 (1.7)	0.0
Large insects >0.5 cm (%)	875 (13.5)	755 (12.7)	704 (12.8)	2,334 (13.0)	101 (12.4)
Small insects <0.5 cm (%)	5,449 (84.3)	5,081 (85.6)	4,720 (86.1)	15,250 (85.3)	717 (87.6)
Total	6,465	5,936	5,482	17,883	818

**Fig. 3.**
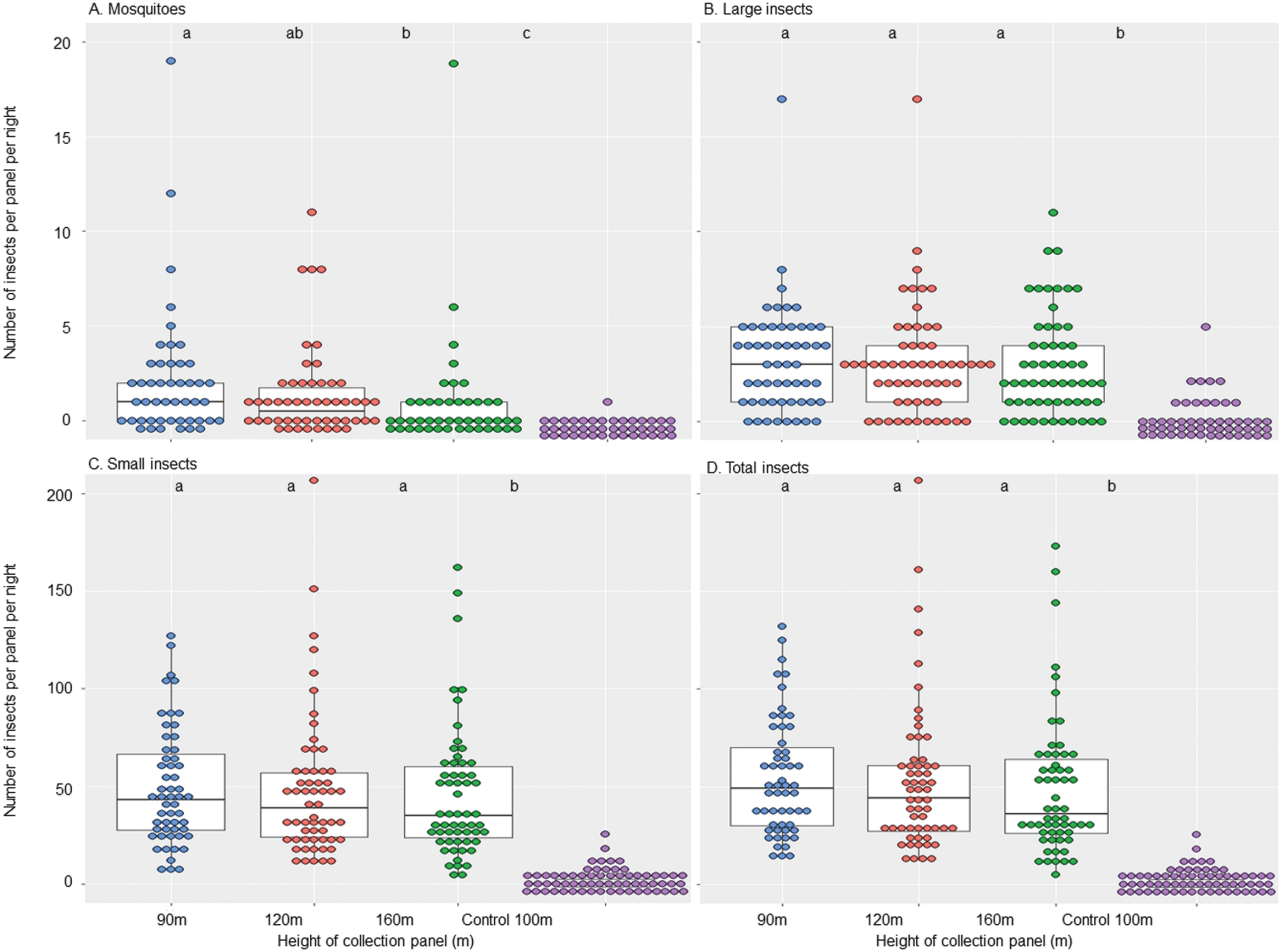
Box plot of insects collected by collection panel height and types of insects. ANOVA post hoc Tukey HSD test by insect types, within each insect type groups not connected by the same letter indicates significantly different from each other.

Collection height significantly affected the number of mosquitoes collected (Kruskal–Wallis, χ^2^ = 12.47, df = 2, *P* = 0.002, Table 1). The number decreased with an increase in panel height, with 1.87 ± 0.44, 1.33 ± 0.30, and 0.50 ± 0.14 mosquitoes/panel/night, respectively, for panels at 90, 120, and 160 m agl, respectively (Fig. 3). In contrast, height agl did not affect the number of small insects or large insects collected (Table 1).

At the same panel height, the average number of individuals collected was always the highest for small insects, followed by large insects, and least for mosquitoes regardless of height (ANOVA, *P* < 0.0001 for all heights). The three insect size groups were significantly different from each other (paired *t*-test, *P* < 0.01 for all pairwise tests). In other words, small insects were dominant regardless of collection height.

### Species Composition of Insects Collected

Further classification of a subset of 2,198 (12%) aerial netted samples demonstrated a diverse composition of insects representing 7 orders (Table 2). Diptera represented the most numerous order of the aerial collections (41.9%), followed, respectively, by Coleoptera (24.2%), Hemiptera, Hymenoptera, Lepidoptera, Odonata, and Neuroptera (<1%). The panel height of 90 m agl consistently netted more insects in 5 orders than panels at higher altitudes.

**Table 2.** Order diversity of collected insects at different heights above the ground

Heights (m)	90	120	160	Total (%)
Diptera	418	295	208	921 (41.9)
Coleoptera	174	169	188	531 (24.2)
Hemiptera	140	181	130	451 (20.5)
Hymenoptera	117	57	47	221 (10.1)
Lepidoptera	12	9	4	25 (1.1)
Odonata	16	4	5	25 (1.1)
Neuroptera	3	1	1	5 (0.2)
Total	888	719	591	2,198

Order compositions were significantly different among the 3 collection heights (χ^2^ = 75.11, df = 12, *P* < 0.0001). For example, at 90 m height, Diptera insects (47.5%) were dominant, whereas at 160 m, Diptera (35.7%) and Coleoptera (32.2%) insects were approximately the same (Table 2).

### Mosquito Diversity at Altitude

A total of 235 morphologically identified mosquito specimens were processed to extract DNA and RNA (Table 3). PCR and COI barcoding assays were successful on 211 (90%) mosquitoes. The possible reasons for missing PCR include false morphological identification due to damaged insect bodies, low-quality DNA extracted, or other species with mutations on primer sites. A sample of 184 specimens were molecularly confirmed as mosquito species by barcoding assay, whereas 27 specimens were classified as other insect species (Table 3; Supplementary Table S1). A total of 7 mosquito genera (*Aedes*, *Anopheles*, *Coquillettidia*, *Culex*, *Mansonia*, *Mimomyia*, and *Aedeomyia*) were identified. The majority were *Culex* (65.8%), followed by *Aedes* (12.5%), and *Coquillettidia* (10.3%). The proportion of *Anopheles* mosquitoes were 5.4% with 5 species that included *Anopheles arabiensis* (4), *Anopheles cf. pharoensis* (2), *Anopheles coustani* (2), *Anopheles leesoni* (1), and *Anopheles maculipalpis* (1). A total of 16 species were classified as *Culex*. The most abundant was *Culex univittatus* (21), followed by *Culex antennatus*, and *Culex neavei*. Six species were identified as *Aedes*, including *Aedes mcintoshi* (15), followed by *Aedes argenteopunctatus*, *Aedes albothorax*, and *Aedes simpsoni*. Note that the lower panel of Table 4 shows a higher number of mosquitoes than the numbers in the higher panels. Among the 184 mosquitoes, none of them was found positive for flaviviruses and alphaviruses by RT-PCR.

**Table 3.** DNA barcode-based molecular identification of mosquito species at high-altitude migration in western Kenya

Genus	*N*	%	Species	Species name^a^ (*n*)
*Anopheles*	10	5.4	5	*Anopheles arabiensis* (4), *Anopheles cf. pharoensis* (2), *Anopheles coustani* (2), *Anopheles leesoni* (1), *Anopheles maculipalpis* (1)
*Culex*	121	65.8	16	*Culex* sp. (24), *Culex univittatus* (21), *Culex antennatus* (18), *Culex neavei* (11), *Culex perexiguus* (8), *Culex rima* (8), *Culex adersianus* (6), *Culex striatipes* (6), *Culex tenagius* (6), *Culex simpsoni* (4), *Culex nebulosus* (2), *Culex sinaiticus* (2), *Culex striatipes* (2), *Culex cinereus* (1), *Culex pipiens* (1), *Culex rubinotus* (1)
*Aedes*	22	12.5	5	*Aedes mcintoshi* (15), *Aedes argenteopunctatus* (3), *Aedes* sp. (2), *Aedes albothorax* (1), *Aedes simpsoni* (1)
*Aedeomyia*	1	0.5	1	*Aedeomyia africana* (1)
*Coquillettidia*	19	10.3	1	*Coquillettidia metallica* (19)
*Mansonia*	10	5.4	2	*Mansonia uniformis* (8), *Mansonia africana* (2)
*Mimomyia*	1	0.5	1	*Mimomyia mimomyiaformis* (1)
Total mosquitoes	184	100.0	31	
Other insects	27		13	*Arrenurus novus* (7), *Procladius dentus* (5), *Chironomus* sp. (3), *Sciaridae* sp. (3), *Arrenurus* sp. (1), *Brevicornu serenum* (1), *Chironomidae sp.* (1), *Chironomus pseudomendax* (1), *Diptera* sp. (1), *Exechia* sp. (1), *Exechiopsis indecisa* (1), *Polypedilum laetum* (1), *Thysanopyga* sp. (1)

^a^sp. represents unnamed species.

**Table 4.** Number of mosquitoes at high-altitude migration in western Kenya (Dec. 2018–Dec. 2019, *n* = 235)

Genus	Panel height			
	90 m	120 m	160 m	Total
*Anopheles*	5	3	2	10
*Culex*	64	51	37	152
*Aedes*	18	12	15	45
*Aedeomyia*	0	0	1	1
*Coquillettidia*	7	6	5	18
*Mansonia*	4	4	0	8
*Mimomyia*	0	0	1	1
Total	98	76	61	235
%	41.7	32.3	26.0	100.0

**Fig. 4.**
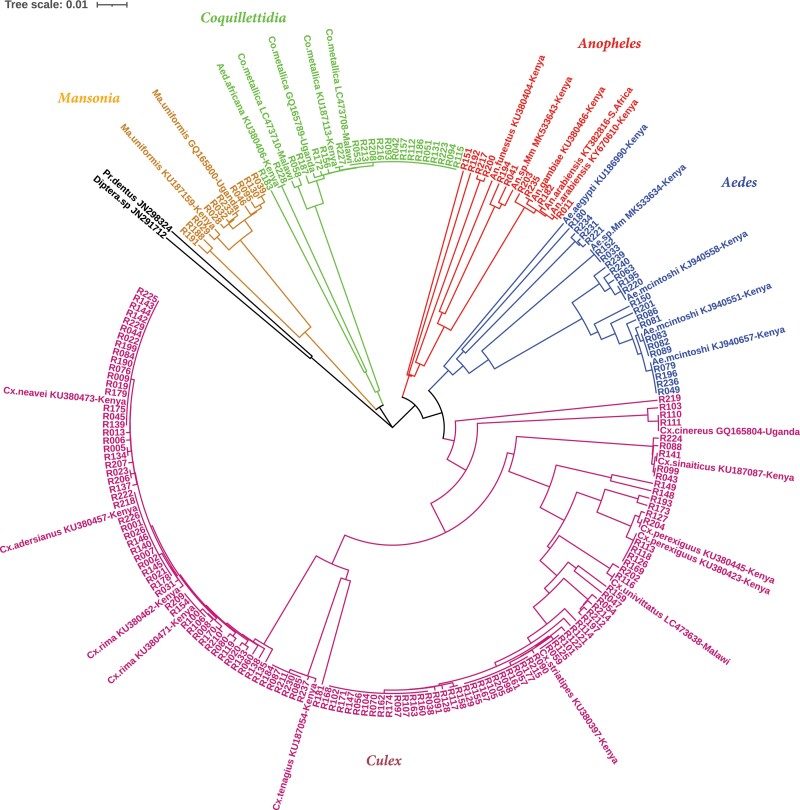
Phylogenetic tree based on barcoding region of COI gene of the specimens at high-altitude migration in western Kenya. A bootstrap resampling analysis was performed (1,000 replicates) to test tree reliability. The tree was rooted to 2 reference sequences (JN298324 and JN291712). Different color represents different genus.

Further analysis of the 184 barcoding sequences using phylogenetic tree clearly showed 6 clusters corresponding to 6 mosquito genera and confirmed species classification and identification of these high-altitude windborne mosquitoes (Fig. 4). The 184 barcoding sequences have been deposited in GenBank under the accession numbers OQ259608–OQ259791.

### Mosquito Survival and Oviposition After High-Altitude Exposure

A total of 310 laboratory-reared colony of *An. gambiae* s.s. females were subjected to the survival assay experiment. Each batch of these females was exposed and observed daily for over a period of 11 days in the month of November–December with varied weather conditions. They were maintained overnight at altitudes of 90, 120, and 160 m agl (Table 5). This design involved the placement of a mosquito in a tube and with netting as described previously (Sanogo et al. 2021). Our results confirmed that the most unfavorable conditions occur in the assay at the high altitude, that is, 160 m agl, likely because wind speed tends to increase with altitude, thus mosquitoes remain trapped in the assay tubes against the wind rather than remaining stationary in relation to the force of air they would be carried by while flying.

**Table 5.** Survival and oviposition rates of *An. gambiae* s.s. across different altitudes

Panel height	*N* replicates	% Survival (*n*)^a^	Oviposition rate (*n*)^a^	Eggs/female^b^
90 m	11	22.7 (25/110)***	80.0 (20/25) n.s.	49.7 ± 8.3 a
120 m	11	17.3 (19/110)***	78.9 (15/19) n.s.	45.1 ± 15.3 a
160 m	11	18.2 (20/110)***	65.0 (13/20) n.s.	35.3 ± 11.9 b
Control	16	85.0 (136/160)	80.0 (24/30)	51.9 ± 10.2 a

^a^χ^2^-test, all tests were against control group. ***Significant at level of 0.001, n.s. not significant at level of 0.05.

^b^ANOVA post hoc Tukey HSD test, numbers connected with different letters indicted significantly different from each other at 0.05 significant level.

The difference in survival was observed relative to control mosquitoes of the same colony that were held in the laboratory insectary and the experimental ones that were held at different altitudes. A survival rate of 85% (*n* = 160) was recorded for the control group, while an overall survival rate of 19% (*n* = 330) was observed in the experimental group (χ^2^ = 194.08, df = 1, *P* < 0.0001). The survival rates were not significantly different among the 3 collection heights (χ^2^ = 1.20, df = 2, *P* = 0.5488) (Table 5). It is worth noting that exposure time was constant across altitudes at 14.5 h overnight and that the tubes and netting material used for holding the mosquitoes up the altitudes on the panels were the same across the experiment.

Oviposition assays were carried out on mosquitoes that survived high-altitude exposures above to assess their ability to lay eggs. Each survivor wass held in an individual 50-ml Falcon tube covered by a netting material and provided with a wet filter paper with little water underneath as a laying pad. The laying pad was examined daily throughout the survival of the individual mosquito. Overall, 75% (*n* = 64) of gravid females subjected to the oviposition assay laid eggs, comparing favorably with the control at 80% (*n* = 30). Due to technical constraints, 25% (*n* = 16) females that did not lay eggs were not dissected immediately for parity and sperm status. Egg counts were done for every individual mosquito. Experimental height affected the average number of egg masses produced by each female, that is, a modestly decreased number of eggs were produced with the increase in height (Table 5).

## Discussion

High-altitude insect aerial sampling conducted in the Sahel region of West Africa (Huestis et al. 2019, Florio et al. 2020, Sanogo et al. 2021) has demonstrated a potential paradigm shift in the mosquito migration as it pertains to public health. Increased knowledge of migration patterns of invertebrates can lead to insights into the changing ecology and dynamics of insect migration (Alerstam et al. 2011, Chapman et al. 2011b, Reynolds et al. 2015). Unlike previous studies of insect migration undertaken in semiarid regions of the Sahel in West Africa, the current high-altitude study was conducted in a midequatorial region of East Africa with high humidity and perennial resources. We observed dispersal activities by multiple insect taxa at high altitude that were similar to those found in aerial collections in West Africa, that is, Diptera, Coleoptera, Hemiptera, and Hymenoptera (Florio et al. 2020). Insect high-altitude migration was found to be a common and widespread phenomenon in the Sahel in accordance with expectations based on the nature of insect habitats (Drake et al. 1995, Chapman et al. 2004, Florio et al. 2020). In contrast, habitat availability and suitability are likely abundant in the area of western Kenya bordering Lake Victoria where the current study was conducted. Interestingly, we observed similar dispersal trends and magnitudes to those observed in the Sahel. In our study, results of a subset sample of approximately 12% of aerial collection identification represented a large number of insects of diverse taxa from hundreds of families across 7 orders. This finding suggests that insect dispersal is a ubiquitous and adaptive behavior, rather than accidental (Baker 1978, 1982). In other words, this modality of dispersal is probably not driven by immediate cues for food, reproduction, or shelter, but is aimed to land the insect in a new habitat with a potential to be more favorable for survival and further breeding than the current one (Gatehouse 1997, Dingle and Drake 2007, Chapman et al. 2015).

The insects sampled in the current study confirmed the notable presence of migratory agricultural insect pests associated with crops of the region, especially rice and maize. For example, we found *Scirpophaga incertulas*, a nocturnal moth (Lepidoptera) that is considered the most damaging insect pest of rice and maize causing yellow stem borer. This pest is characterized with longitudinal white patches on leaf sheaths, central leaf whorl drying, and brown discolorations that eventually lead to drying of the tiller without the production of the panicles that produce grains. Other rice pests that were found in this study include *Parapoynx stagnalis stagnalis* (Lepidoptera), a common rice case worm whose larva scrapes chlorophyl II from leaves, resulting in wilting at immature stage, *Orseolia oryzea* (Diptera), a rice gall midge that bores into rice bud during the tillering stage. Similar findings were also reported from the Sahel of West Africa where an assortment of windborne migrants that transmit or cause human, animal, and plant disease outbreaks was found (Florio et al. 2020). The presence and diversity of these high-altitude migratory insects in both perennially resource-endowed (Kenya) and seasonal areas (Sahel) confirm the fact that migration is a deliberate individual behavior specific to particular insects. It is characterized as a persistent flight by insects to a new environment (Kennedy 1985, Dingle and Drake 2007, Dingle 2014, Chapman et al. 2015), whereas non-migratory flights are generally driven and terminated by search for and encounter with specific resources such as food, a mate, an oviposition site, or just suitable habitat (Sappington et al. 2018). Therefore, more than simply escaping deteriorating environmental conditions, however, migratory flight behavior is usually directional and designed to move the insect to an area where more favorable breeding conditions prevail (Chapman et al. 2011a). Many pests of field crops are migratory species that fit this description (Magor 1995, Drake and Reynolds 2012).

Over the past 5 decades, efforts to confirm viability postmigration in many high-altitude windborne migrant insects have been established by studies comparing survival and oviposition in alive collection of insects such as small diptera with those captured on the ground or by simulated long flights (Cockbain 1961, Mcanelly and Rankin 1986). For decades, some biologists considered high-altitude windborne migration in mosquitoes to be accidental and thus of negligible significance (Service 1997) and therefore doubt the viability of the mosquitoes collected at high altitude given the prevailing unfavorable conditions. Recently, a systematic sampling of insects that migrate at high altitude found that multiple malaria vectors engage in high-altitude wind-assisted movements across seasons and that they are likely to carry *Plasmodium* given that majority were found to be gravid (Huestis et al. 2019). Consequently, further studies in the Sahel demonstrated that mosquitoes are able to withstand high-altitude flight like other migratory insects and subsequently reproduce and transmit pathogens by blood feeding on new host (Sanogo et al. 2021). The current experiment on survivorship and oviposition of laboratory-reared *An. gambiae* s.s. at high altitude was carried out during rainy season characterized by low RH and high wind speeds—conditions known to reduce mosquito survival (Clements 1992, Huestis and Lehmann 2014, Arcaz et al. 2016, Sanogo et al. 2021). Despite these overnight harsh environmental and survival assays conditions similar to previous experiment by Sanogo et al. (2021), a moderate proportion of the mosquitoes survived compared with the controls held in the laboratory. Subsequently, the majority of surviving mosquitoes subjected to oviposition assays demonstrated a minimal difference in egg batch laying capacity and viability compared to their control counterparts that were maintained in the laboratory. Furthermore, the results have confirmed that similar to previous mosquito studies (Sanogo et al. 2021) and other migratory insect species that have been evaluated (Taylor 1960, Cockbain 1961, Mcanelly and Rankin 1986), mosquitoes are able to survive high-altitude flight and subsequently reproduce. Together with the evidence of high-altitude migration of malaria vectors in West Africa, the findings from this study suggest that such a windborne dispersal activity of malaria vectors may occur across sub-Saharan Africa and thus, has implication on transfer of pathogens, insecticide-resistant genes, in insects of importance to public health and food security. These unique similarities and distinction in aerial insect dominance between far apart and environmentally diverse regions clearly call for further investigation to identify possible genetic linkages and the specific cues that trigger such phenomenon movement at high altitude.

## Supplementary Material

tjad033_suppl_Supplementary_Table_S1
